# Sample Size under Inverse Negative Binomial Group Testing for Accuracy in Parameter Estimation

**DOI:** 10.1371/journal.pone.0032250

**Published:** 2012-03-22

**Authors:** Osval Antonio Montesinos-López, Abelardo Montesinos-López, José Crossa, Kent Eskridge

**Affiliations:** 1 Facultad de Telemática, Universidad de Colima, Colima, Colima, México; 2 Departamento de Estadística, Centro de Investigación en Matemáticas (CIMAT), Guanajuato, Guanajuato, México; 3 Biometrics and Statistics Unit, Maize and Wheat Improvement Center (CIMMYT), Mexico D.F., Mexico; 4 Department of Statistics, University of Nebraska, Lincoln, Nebraska, United States of America; National University of Ireland Maynooth, Ireland

## Abstract

**Background:**

The group testing method has been proposed for the detection and estimation of genetically modified plants (adventitious presence of unwanted transgenic plants, AP). For binary response variables (presence or absence), group testing is efficient when the prevalence is low, so that estimation, detection, and sample size methods have been developed under the binomial model. However, when the event is rare (low prevalence <0.1), and testing occurs sequentially, inverse (negative) binomial pooled sampling may be preferred.

**Methodology/Principal Findings:**

This research proposes three sample size procedures (two computational and one analytic) for estimating prevalence using group testing under inverse (negative) binomial sampling. These methods provide the required number of positive pools (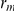), given a pool size (*k*), for estimating the proportion of AP plants using the Dorfman model and inverse (negative) binomial sampling. We give real and simulated examples to show how to apply these methods and the proposed sample-size formula. The Monte Carlo method was used to study the coverage and level of assurance achieved by the proposed sample sizes. An R program to create other scenarios is given in Appendix S2.

**Conclusions:**

The three methods ensure precision in the estimated proportion of AP because they guarantee that the width (*W*) of the confidence interval (CI) will be equal to, or narrower than, the desired width (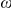), with a probability of 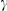. With the Monte Carlo study we found that the computational Wald procedure (method 2) produces the more precise sample size (with coverage and assurance levels very close to nominal values) and that the samples size based on the Clopper-Pearson CI (method 1) is conservative (overestimates the sample size); the analytic Wald sample size method we developed (method 3) sometimes underestimated the optimum number of pools.

## Introduction

To detect the presence of a rare event, thousands of individuals need to be tested, and the cost of such testing usually exceeds the available budget and staff. The pooling methodology (Dorfman method) was first proposed to save a significant amount of money when detecting soldiers with syphilis [1]. Significant cost savings were achieved by first testing a sample created by mixing blood from several people. If the sample tested positive, the blood from each individual in that pool would be retested; if the sample tested negative, all individuals in that pool were declared free of the disease [1]. Currently the Dorfman method is used for detecting and estimating the proportion of positive individuals in fields such as medicine [2], [3], [4], [5], agriculture [6], telecommunications [7], and science fiction [8]. Most applications for detecting and estimating a proportion are developed using binomial sampling; however, Pritchard and Tebbs [9] have suggested that inverse (negative) binomial pooled sampling may be preferred when prevalence *p* is known to be small, when sampling and testing occur sequentially, or when positive pool results require immediate analysis—for example, in the case of many rare diseases. Unlike binomial sampling, in this model the number of positive pools to be observed is fixed *a priori*, and testing is complete when the rth positive pool is reached [10].

George and Elston [11] recommended using geometric sampling when the probability of an event is small; they gave confidence intervals for the prevalence based on individual testing. Also, according to Haldane [12], using a binomial distribution may not provide an unbiased and precise estimate of *p* when *p* is small (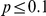). Lui [13] extended George and Elston's work [11] on the confidence interval (CI) by considering negative binomial sampling and showed that as the required number of successes increased, the width of the CI decreased. However, this extension was also under individual testing. Using negative binomial group testing sampling, Katholi [14] derived point and interval estimators of *p*, obtained by both classical and Bayesian methods, and investigated their statistical properties.

Recently Pritchard and Tebbs [9] used maximum likelihood as a basis for developing three point and interval estimators for *p* under inverse pooled sampling; they compared its performance with Katholi's [14] proposed point and interval estimators. Pritchard and Tebbs [10] extended their work to Bayesian point and interval estimation of the prevalence under negative binomial group testing. They used different distributions to incorporate prior knowledge of disease incidence and different loss functions, and derived closed-form expressions for posterior distributions and point and credible interval estimators [10]. However, until now sample size procedures under inverse (negative) binomial sampling for group testing have not been proposed.

In practice, pooling is a simple process; for example, if 40,000 plants are collected from the field, they could be tested one at a time for detecting unwanted transgenic plants (AP). If each test takes 15 minutes and costs US$12, then this project will take 10,000 hours and cost US$480,000. A shorter approach would be to smash 10 plants together and test this pooled sample [15]. This approach would take 1000 hours and cost US$48,000. Even greater savings are achieved with larger pool sizes. However, because the maximum likelihood estimator (MLE) of *p* under binomial [16] and negative binomial [9], [10] group testing is biased to the right, then, on average, the MLE of *p* overestimates the true prevalence for any pool size (assuming a perfect diagnostic test); however, this bias is usually small when *p* is small (*p*<0.1) [17]. In addition, if the diagnostic test is imperfect, a high rate of false positives is very likely. Thus, there are benefits and risks attached to the use of pooling methodology [15]. For this reason, it is important to choose the pool size with care in order to guarantee precision in the estimation process.

Under binomial group testing, some authors have proposed methods for determining the required sample size (number of required pools) to guarantee a certain level of power and/or precision [18], [19], [20], [21]. Yamamura and Hino [18] and Hernández-Suárez et al. [19] developed sample size methods in terms of power considerations. This approach is consistent with the emphasis on hypothesis testing for inference, with results reported in terms of *p*-values. Montesinos-López et al. [20], [21] developed sample size procedures under the *accuracy in parameter estimation* (AIPE) framework that guarantee narrow confidence intervals for estimating the parameter. The use of this approach is increasing, not only because the CIs ensure that the magnitude of the effect can be better assessed, but also because the effect in question can be readily identified by the reader. Furthermore, CIs also convey information about how precisely the magnitude of the effect can be ascertained from the data at hand [22]. Another advantage of the AIPE approach is that it treats the estimates (from pilot studies or literature review) used to determine the required sample size as random to guarantee that the desired CI width for estimating the parameter of interest is achieved, as originally planned [23].

However, under binomial group testing sampling when the prevalence is low, the calculated sample size sometimes does not contain any pools with the trait of interest (i.e., failure to detect and estimate AP). For this reason, inverse (negative) binomial sampling is a good alternative because each sample will contain the desired number of rare units and also the sample size is not a fixed quantity [12], [9], [10]. In binomial group testing, the number of required pools is treated as a fixed quantity, whereas under inverse (negative) binomial group testing, the pools are drawn one by one until the sample contains exactly 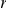 positive pools (here the number of positive pools is fixed).

Based on the previous findings, the purpose of the present study is to develop methods for determining sample size (number of positive pools) under inverse (negative) binomial group testing with the objective of increasing accuracy in the estimation of the population proportion. This research proposes methods for determining the required number of positive pools, with the aim of estimating the proportion of AP (*p*) using inverse (negative) binomial group testing with a perfect test and fixed pool size (*k*) that will assure a narrow CI. Accuracy in the estimation of *p* is achieved because CI width is considered stochastic and thus treated as a random variable. The methods used for achieving the objectives of the present research are: point and interval estimation for the population proportion, delta method, and central limit theorem. We provide an R program that reproduces the results presented in this study and makes it easy for the researcher to create other scenarios.

## Materials and Methods

Suppose that 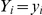 represents the number of pools tested until the first positive pool is detected and 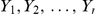 are observed to obtain the 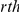 positive pool. Therefore, 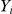 has a geometric distribution. Therefore, the overall number of pools that are tested to find *r* positive pools is equal to 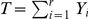. In what follows, we shall denote the size of the pools collected as *k* and assume equal pool size; the prevalence of infection is denoted by *p*, the number of pools tested to find one positive pool is 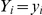, and the number of times this experiment is carried out is denoted by *r*. It is important to mention that in this paper we consider that: (i) the sample size is the value of *r* that represents the number of positive pools required to stop the sampling and testing process, and (ii) the overall number of pools tested is the value of 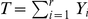. If the prevalence of infection is *p*, then the probability that a pool of size *k* tests positive is 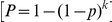. Therefore, the sufficient statistics 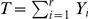 follows a negative binomial distribution (nib) with waiting parameter *r* and success probability 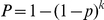 [9], [10], [14]. According to Pritchard and Tebbs [9], [10] and Katholi [14], the maximum likelihood estimate (MLE) of 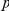 using inverse (negative) binomial group testing is
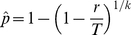
(1)where *k* is the pool size and 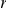 is the fixed required number of positive pools. This MLE of *p* for inverse (negative) binomial group testing with groups of equal size assumes a perfect diagnostic test. On the other hand, the variance of 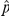 according to Pritchard and Tebbs [9], [10] and Katholi [14] is given by 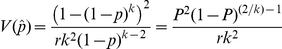. According to Pritchard and Tebbs [9], the corresponding Wald CI is as follows:
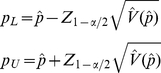
(2)where 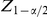 is the 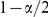 quantile of the standard normal distribution, and 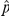 is the MLE estimated from Eq. (1). This approximation of the CI is easy to calculate and allows deriving closed-form sample size formulas. However, when 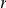 is small, the normal approximation for MLE is doubtful; in such cases, the Wald-type CI often produces negative endpoints. In addition, the coverage probability of the CIs constructed by Wald-type CIs is often smaller than 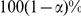.

### Derivation of the sample size formula for detecting transgenic plants

The quantity 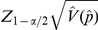 (added and subtracted from the observed proportion, 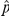) in Eq. (2) is defined as *W/2* (where *W* is the full width of the CI; *W* or *W/2* can be set *a priori* by the researcher depending on the desired precision). The observed CI width for any realization of a confidence interval (from Eq. 2) can be expressed as:
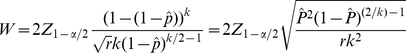
(3)Let 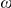 be the desired CI width; then the basic AIPE approach seeks to find the minimum sample size so that the expected CI width is sufficiently narrow [24], [25]. In other words, the AIPE approach seeks the minimal sample size so that 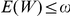. The problem is that the expected CI width is an unknown quantity, although it can be approximated. As 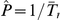, where 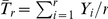, the observed width, *W*, is a function of 
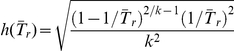
. Since the distribution of 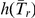 is unknown, it is not possible to obtain an analytic solution for 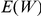. An alternative is to use the delta method to derive the asymptotic distribution of 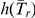. From Result 1 in Appendix S1, we have that
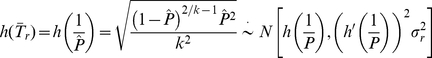
where 
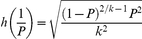
, 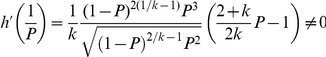 for 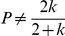. Therefore, the expected value of *W* is 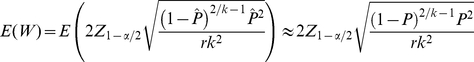. Now if we set the 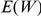 to the desired width of the CI, 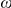:
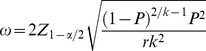
(4)Solving for 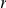, Eq. (4) yields the following formulation:
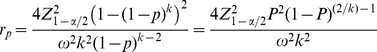
(5)Note that if 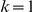, Eq. (5) reduces to the formula derived by Lui [13] 
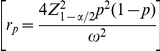
. However, Eq. (5) requires the population value of 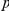, which is unknown and in practice is replaced by an estimation of the true proportion. Eq. (5) finds the required sample size for achieving an expected CI width, 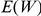, that is sufficiently narrow for estimating the proportion of AP using pools; however, this does not guarantee that for any particular CI, the observed expected CI width, 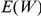, will be sufficiently narrow, because the expectation only approximates the mean CI width. Kelley and Rausch [25] state that this issue is similar to the case where a mean is estimated from a normal distribution; although the sample mean is an unbiased estimator of the population mean, the sample mean will almost certainly be smaller or larger than the population value. This is because the sample mean is a continuous random variable, as is the CI width, due to the fact that both are based on random data. Thus, approximately half of the time, the computed confidence interval will be wider than the desired (specified) width [25].

Since Eq. (3) uses an estimate of 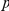 ^,^ the CI width (*W*) is a random variable that will fluctuate from sample to sample. This implies that, using 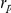 from Eq. (5), less than 50% of the sampling distribution of *W* will be smaller than 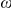 (see the third column in Table 1). To demonstrate this, we need to calculate the probability of obtaining a CI width that is smaller than the specified value (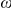). This can be computed as:
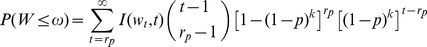
where 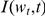 is an indicator function showing whether or not the actual CI width calculated using Eq. (3 ) is ≤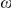, 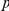 is the true population proportion and 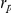 is the sample size obtained using equation (5). To avoid possible computer limitations, the above probability can be approximated by the following:
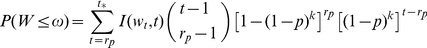
(6)where 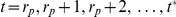, and 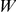 is considered a random variable because the exact value of *p* is not known and 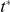 is the value that satisfies 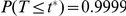; we use this value of 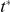 because in the R package summing to infinity is not possible.

**Table 1:** Underestimation of the sample size given by using Eq. (5) (Table 1A).

A	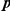	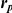	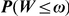	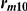	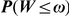	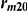	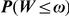	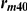	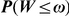
	0.005	8	0.4602923	18	0.9439192	28	0.9985824	48	0.9999997
	0.0075	18	0.4937528	28	0.8677739	38	0.9860621	58	0.9999798
	0.01	31	0.4764102	41	0.792025	51	0.9491423	71	0.9993324
	0.0125	49	0.4825564	59	0.7531049	69	0.9091713	89	0.9962656
	0.015	70	0.4831282	80	0.6966756	90	0.867216	110	0.9873122
	0.0175	96	0.49556	106	0.6823066	116	0.83486	136	0.9736274
	0.02	126	0.4923463	136	0.6682315	146	0.8073307	166	0.9575451
	0.0225	159	0.4885201	169	0.6302238	179	0.7655083	199	0.9288043
	0.025	198	0.5028085	208	0.631837	218	0.7583371	238	0.9121938

B	*k*	*r* = 5	*r* = 15	*r* = 25	*r* = 35	*r* = 45	*r* = 55	*r* = 65	*r* = 75
	5	0.562	0.535	0.528	0.523	0.520	0.519	0.518	0.515
	15	0.566	0.539	0.529	0.524	0.522	0.518	0.517	0.516
	25	0.561	0.536	0.529	0.524	0.523	0.513	0.512	0.513
	35	0.573	0.539	0.528	0.520	0.518	0.515	0.514	0.516
	45	0.550	0.537	0.522	0.524	0.516	0.521	0.512	0.517
	55	0.549	0.543	0.522	0.527	0.516	0.521	0.514	0.517
	65	0.533	0.520	0.515	0.514	0.512	0.512	0.514	0.513
	75	0.530	0.520	0.518	0.521	0.517	0.518	0.517	0.520
	85	0.568	0.548	0.542	0.514	0.514	0.517	0.519	0.522

C	*k*	*r* = 5	*r* = 15	*r* = 25	*r* = 35	*r* = 45	*r* = 55	*r* = 65	*r* = 75
	5	1.4E-05	2.3E-06	1.2E-06	8.2E-07	6.2E-07	5.0E-07	4.1E-07	3.5E-07
	15	2.4E-05	2.4E-06	1.2E-06	8.2E-07	6.3E-07	4.9E-07	4.1E-07	3.5E-07
	25	3.4E-05	2.3E-06	1.2E-06	8.2E-07	6.2E-07	4.9E-07	4.1E-07	3.6E-07
	35	1.4E-04	2.3E-06	1.2E-06	8.2E-07	6.1E-07	5.0E-07	4.2E-07	3.5E-07
	45	3.1E-04	2.4E-06	1.2E-06	8.3E-07	6.2E-07	5.0E-07	4.1E-07	3.6E-07
	55	8.9E-04	2.4E-06	1.2E-06	8.4E-07	6.2E-07	5.0E-07	4.2E-07	3.6E-07
	65	1.6E-03	2.4E-06	1.2E-06	8.2E-07	6.2E-07	5.0E-07	4.2E-07	3.6E-07
	75	2.7E-03	2.4E-06	1.3E-06	8.3E-07	6.3E-07	5.1E-07	4.2E-07	3.6E-07
	85	5.1E-03	2.4E-06	1.3E-06	8.3E-07	6.4E-07	5.1E-07	4.2E-07	3.6E-07

Table 1A. Preliminary sample size (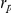, number of required positive pools) for estimating the population proportion, computed with Eq. (5) and three sample size increments (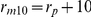, 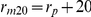, and 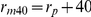) with their corresponding probability that the confidence interval width 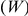 is smaller than the specified value (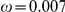), 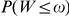 computed with Eq. (6). For a 95% CI and 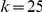, 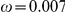 is the desired CI width. 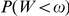 is the probability that (*W*) is smaller than the specified value (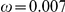) calculated using Eq. (6). Table 1B. Proportion of times the MLE of *p* is greater than the population proportion 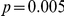 for different combinations of values of 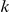 and 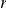 that produce simulated 40, 000 samples. Table 1C. Mean Square Error for 40, 000 simulated samples with 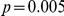 and different values of 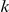 and 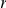.

### Degree to which the sample size is underestimated using Eq. 5

To show the degree to which 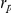 is underestimated using Eq. (5), we give an example (Table 1A) in which Eq. (6) is used to calculate 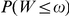, that is, the probability that *W* will be smaller than, or equal to, the desired CI width (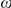) for a given value 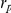 (number of positive pools) obtained using Eq. (5). The numerical example in Table 1 is given for several values of the population proportion (*p*) for a CI of 95%, 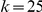, and for a desired width of 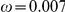. Table 1A presents the preliminary sample size 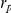 computed with Eq. (5), and three other increments computed as 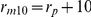, 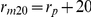, and 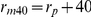. For each sample size, the probability that *W* is smaller than the specified value (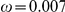), 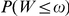, is calculated using Eq. (6). This is done to show that the required number of positive pools for the proportion (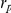, second column in Table 1A) computed using Eq. (5) has a probability of around 0.50 that 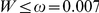 (third column in Table 1A). For example, when 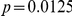, the preliminary sample size (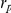) is 49 and the probability of obtaining a *W* 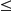 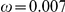 is 0.4825564. With 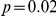, 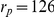, we can only be 49.235% certain that *W* will be 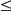 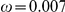. When the number of pools increases by 10 (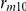, fourth column, Table 1A) or by 20 (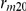, sixth column, Table 1A), the probability 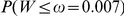 increases. For example, when 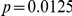, there are 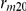 = 69 units (pools) in the sample with 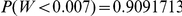; for 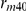 = 89 pools in the sample, the 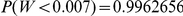. Thus, results of Table 1A show that in order to ensure a high 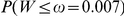, a bigger sample size (number of positive pools) than the preliminary one (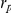) calculated using Eq. (5), is required. Also, we see in Table 1A that 8 times out of 9 the preliminary sample size (number of positive pools) resulting from using Eq. (5) produces a 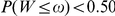, that is, 88.89% of the time *P*(*W* 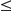 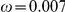) was lower than 50%.

For 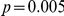, and a different combination of values of *k* and *r* that produces 40,000 samples, Table 1B shows that for larger values of *r*, the percentage of times that the MLE of *p* is larger than the population proportion is lower. These results also show that the level of underestimation of the required number of pools (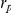) caused by the use of Eq. (5) is important and is mainly due to the fact that half of the time the population proportion 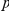 will be lower than the estimated proportion 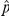 (Table 1B)^;^ thus the obtained CI width (*W*) will be larger than the specified 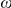 about more than half of the time. However, the expected value of the computed *W* is the value specified *a priori* (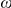), provided the correct value of the population variance is used. Therefore, the use of Eq. (5) will ensure that the desired width 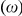 for the CI will be obtained less than 50% of the time, that is, 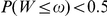. The values of the Mean Square Error (MSE) for 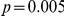 and different combinations of *k* and *r* (Table 1C) indicate MSE increases for lower values of *r*, however, no values of *k* seem to guarantee low bias.

Since Eq. (5) underestimates the required number of pools, in the following section, we propose three new methods to estimate the optimum sample size (two computational and one analytic).

### Computational optimum sample size estimation–methods 1 and 2

The optimal sample size is the smallest integer value (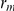) such that
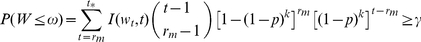
(7)where 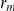 will start with a minimal sample size, say 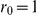, and 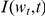 is an indicator function showing whether or not the actual CI width (*W*) is ≤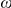. The CI width will be calculated as 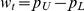. We determined that method 1 is when an exact 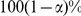 CI for 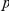 is used, and method 2 is when the CI is computed using the Wald CI (Eq. 2) and Eq. (7), which we call the computational Wald procedure.

The CI used for the exact method (method 1) is the Clopper-Pearson CI, as explained in the following. When equal pool sizes *k* are used, 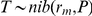, where 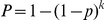. Using the relationship between the negative binomial distribution and the incomplete beta function, Lui [13] derived an exact interval for 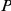. The lower and upper confidence limits are 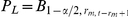 and 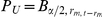, respectively, where 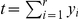 and 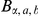 denotes the 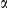 quantile of the two-parameter beta distribution [9]. Thus an exact 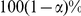 CI for *p* can be obtained by suitably transforming the endpoints of the 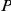 interval, i.e., 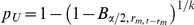 and 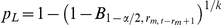 [9]. Also, this interval for *p* can be formed using the relationship between the negative binomial and F distribution, in this case 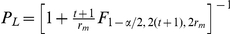 and 
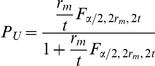
, where 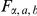 denotes the upper 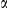 quantile of the two-parameter F distribution. Again, an exact 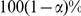 CI for *p* is 
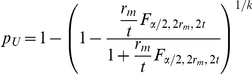
 and 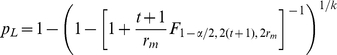 [26]. This last version of the Clopper-Pearson CI has the advantage that the exact CI for *p* can be calculated by hand using standard F tables.

In methods 1 and 2, we start with a minimal sample size, say 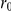, and increase the initial number of pools (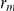) by one unit, recalculating Eq. (7) each time, until the desired degree of certainty (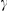) is achieved; this will produce a modified number of pools (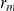) that assures, with a probability 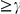, that the *W* will be no wider than 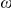. In other words, 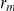 ensures that the researcher will have approximately 100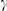 percent certainty that the computed CI will have the desired width or smaller. For example, if the researcher requires 90% confidence that the obtained *W* will be no larger than the desired width (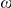), (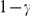) would be defined as 0.10, and there would be only a 10% chance that the CI width, around 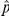, would be larger than specified (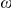) [24], [27].

Contrary to Eq. (5) above, the computational sample size proposed by Eq.(7) with methods 1 and 2 considers 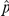 as a random variable and gives a non-closed-form solution for computing a minimum sample size (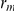) that guarantees that *W* is smaller than, or equal to, 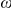 with a probability of at least 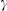. In the following section, we propose a closed-form analytic method for determining the optimal sample size (number of positive pools required) that uses a single formula which assures the estimation of a narrow confidence interval.

### Analytic optimum sample size estimation–method 3

The CI width using the Wald interval for *p* is 
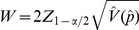
, and *W* must be smaller than a specified value (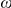) with probability (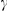). Therefore, the optimal sample size is defined as being the smallest integer value (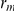) such that
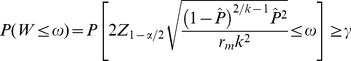
(8)From Result 2 in Appendix S1, for fixed 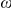, the number of required positive pools with method 3 is given by
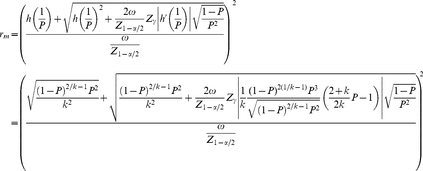
(9)where 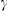 represents the desired degree of certainty (required probability) of achieving a CI width (*W*) for 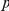 that is no wider than the desired value (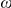). 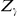 is the 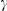 quantile of the standard normal distribution. 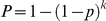 is the probability of a positive pool. Note that if 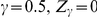 (because the 50% quantile of a standard normal distribution is required), then Eq. (9) reduces to Eq. (5), that is, the formula determines the required number of pools assuming that the proportion of the population 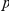 is known and fixed; this means, as already anticipated, that the required width *W* will be achieved only 50% of the time approximately. On the other hand, if 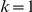, Eq. (9) reduces to
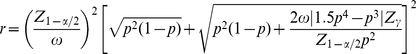
(10)which is appropriate for determining the sample size without grouping (without making pools) (individual testing because *k* = 1) and guarantees that *W* will be smaller than, or equal to, 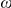 with a probability 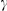. In other words, only 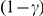 of the time will *W* be larger than the desired CI width, 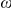.

Also note that when 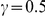, Eq. (10) [individual inverse (negative) binomial sample size] reduces to the formula proposed by Lui [13] under individual inverse (negative) binomial sampling, 
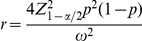
 when the stochastic nature of the CI width is not considered. It is important to point out that Eq. (7) and the proposed formulas Eq. (9) and (10) determine a minimum sample size (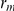) that guarantees that *W* will be smaller than, or equal to, 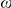 with a probability of at least 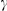. In contrast to Eq. (5), Eqs. (7), (9), and (10) account for the stochastic nature of the random variable 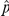 via the desired degree of certainty (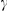). It should be pointed out that 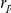 is what we call the sample size obtained from Eq. (5) or from Eq. (9) or (7) using 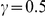, and 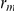 is the sample size obtained with Eq. (9) or (7) when 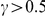. For this reason, the level of assurance would be 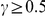. When using Equations (9) or (7), we suggest three ways of specifying the value of *p*: (1) perform a pilot study, (2) use the value of *p* reported in the literature of similar studies, and (3) use the upper bound for *p* that was reported. The upper bound should be chosen carefully to avoid estimators with high bias and high MSE; also, the upper bound needs to be used when the study was performed under group testing and when the value of *r* is not small [9]. In addition, if the value of *p* reported in the literature was not obtained using group testing (but rather individual testing), then using an upper bound for sample size determination is not recommended. On the other hand, it is important to point out that the sample size from Equation (5) or from Equation (7) or (9) when using 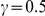 will be called preliminary sample size in order to distinguish it from the sample size obtained from Equations (7) or (9) when level 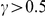.

## Results

Sample sizes are shown for *k* values of 40 (Table 2), *p* values ranging from 0.005 to 0.025, and 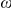 values from 0.007 to 0.010 by 0.001 for each method. Within this table, we delineated three sub-tables with the modified number of pools 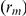 and 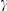 values of 0.50, 0.80, and 0.90, each for a CI coverage of 95%. Each condition is crossed with all other conditions in a factorial manner; thus there are a total of 108 different cases for planning an appropriate sample size for each proposed method. To examine the results shown in Table 2, a simulation study was performed to examine the coverage and assurances of the samples as compared with the nominal coverage and assurances [Table 3 for the analytic procedure (method 3); Table 4 for the computational Wald procedure (method 2), and Table 5 for the exact Clopper-Pearson procedure (method 1)].

**Table 2:** Sample size (required number of positive pools) for the three methods^b^.

	Analytic formula (method 3)				Clopper-Pearson (method 1)				Computational Wald (method 2)			
	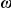	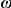	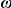			
*p*	0.007	0.008	0.009	0.010	0.007	0.008	0.009	0.010	0.007	0.008	0.009	0.010
	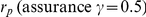				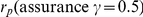				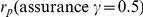			
0.005	8	6	5	4	9	7	6	5	9	7	6	5
0.0075	18	14	11	9	19	15	12	10	19	15	12	10
0.01	31	24	19	15	34	26	21	17	33	25	20	17
0.0125	49	38	30	24	52	41	33	27	50	39	31	25
0.015	72	55	43	35	75	59	46	38	73	56	45	36
0.0175	98	75	59	48	103	80	63	52	100	76	61	50
0.02	130	99	78	64	136	105	84	68	131	101	80	65
0.0225	166	127	101	81	174	134	106	86	168	128	101	82
0.025	208	159	126	102	218	167	133	109	209	160	126	104
	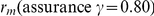				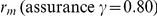				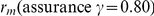			
0.005	12	10	8	7	14	12	10	9	14	12	10	8
0.0075	24	19	16	13	26	22	18	15	26	21	17	15
0.01	40	32	26	22	44	35	29	24	43	33	28	24
0.0125	61	48	39	32	65	52	43	35	63	50	40	34
0.015	86	67	54	45	91	71	59	49	88	69	56	47
0.0175	115	90	72	60	121	96	77	65	118	93	75	62
0.02	149	116	94	77	156	123	100	82	151	118	96	80
0.0225	189	147	118	97	198	154	126	104	190	150	120	99
0.025	234	182	146	120	244	191	154	128	237	185	148	122
	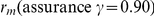				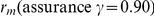				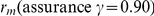			
0.005	14	11	9	8	17	14	12	11	17	14	12	11
0.0075	27	22	18	15	31	25	21	18	30	25	21	18
0.01	45	36	29	25	49	39	33	29	48	38	32	27
0.0125	67	53	43	36	71	57	48	40	70	56	46	39
0.015	93	73	59	50	98	79	65	55	96	76	62	53
0.0175	123	97	79	65	130	104	85	71	127	101	82	69
0.02	159	125	101	84	167	134	109	91	163	128	105	87
0.0225	200	157	127	105	211	166	136	113	203	160	131	110
0.025	247	193	156	129	260	205	167	138	250	197	159	132

b For a CI of 95%, 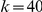, four desired widths (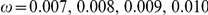) and three values of 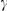 (0.5, 0.8, and 0.90). The value of 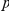 is the population proportion, 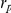 is the preliminary number of required positive pools, 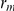 is the modified required number of positive pools, and 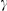 is the assurance for the desired degree of certainty of achieving a CI for 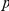 that is no wider than the desired CI width (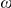).

**Table 3:** Simulation study of the coverage and assurance for method 3 (analytic formula)^c^.

	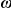	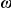
*P*	0.007	0.008	0.009	0.010	0.007	0.008	0.009	0.010
	-------Coverage 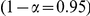 --------				----------Assurance 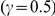 -----------			
0.0050	0.9550	0.9553	0.9590	0.9534	0.4670	0.4323	0.4764	0.4543
0.0075	0.9530	0.9585	0.9534	0.9573	0.4917	0.4782	0.4863	0.4613
0.0100	0.9512	0.9546	0.9508	0.9555	0.4573	0.4713	0.4669	0.4546
0.0125	0.9508	0.9518	0.9551	0.9522	0.4601	0.4973	0.4920	0.4787
0.0150	0.9497	0.9475	0.9527	0.9541	0.4886	0.4731	0.4485	0.4614
0.0175	0.9513	0.9506	0.9533	0.9533	0.4821	0.4696	0.4826	0.4895
0.0200	0.9525	0.9516	0.9539	0.9523	0.4867	0.4835	0.4893	0.4826
0.0225	0.9483	0.9527	0.9458	0.9539	0.4949	0.4878	0.5046	0.4850
0.0250	0.9527	0.9514	0.9481	0.9472	0.5019	0.4907	0.4992	0.4725
	------Coverage 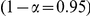 -------				---------Assurance 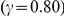 -----------			
0.0050	0.9521	0.9542	0.9546	0.9581	0.7314	0.7523	0.7352	0.7334
0.0075	0.9546	0.9571	0.9549	0.9542	0.7367	0.7324	0.7626	0.7191
0.0100	0.9509	0.9515	0.9534	0.9548	0.7603	0.7573	0.7538	0.7743
0.0125	0.9489	0.9557	0.9495	0.9494	0.7725	0.7594	0.7622	0.7653
0.0150	0.9511	0.9488	0.9525	0.9520	0.7819	0.7704	0.7536	0.7839
0.0175	0.9521	0.9538	0.9499	0.9511	0.7760	0.7781	0.7630	0.7678
0.0200	0.9484	0.9495	0.9507	0.9493	0.7780	0.7692	0.7740	0.7522
0.0225	0.9491	0.9514	0.9541	0.9495	0.7848	0.7636	0.7766	0.7656
	-------Coverage 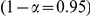 -------				---------Assurance 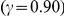 -----------			
0.0050	0.9535	0.9524	0.9551	0.9546	0.8300	0.8007	0.7798	0.8127
0.0075	0.9504	0.9534	0.9527	0.9532	0.8434	0.8537	0.8385	0.8301
0.0100	0.9502	0.9503	0.9521	0.9508	0.8741	0.8686	0.8384	0.8583
0.0125	0.9534	0.9495	0.9539	0.9552	0.8689	0.8672	0.8483	0.8580
0.0150	0.9476	0.9545	0.9510	0.9501	0.8670	0.8722	0.8646	0.8677
0.0175	0.9515	0.9538	0.9543	0.9521	0.8757	0.8682	0.8633	0.8570
0.0200	0.9490	0.9484	0.9487	0.9549	0.8781	0.8723	0.8764	0.8644
0.0225	0.9490	0.9500	0.9520	0.9544	0.8766	0.8767	0.8850	0.8744
0.0250	0.9522	0.9488	0.9543	0.9492	0.8803	0.8671	0.8784	0.8698

c These coverages and these levels of assurance are for sample sizes obtained with the analytic formula (method 3) presented in Table 2, for a CI of 95%, 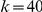, four desired widths (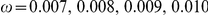), and three values of assurance 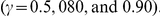

**Table 4:** Simulation study of coverage and assurance for method 2^d^.

	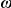	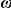
*p*	0.007	0.008	0.009	0.010	0.007	0.008	0.009	0.010
	-------Coverage 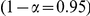 --------				----------Assurance 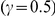 -----------			
0.0050	0.9544	0.9580	0.9538	0.9581	0.5393	0.5431	0.5653	0.5498
0.0075	0.9548	0.9523	0.9533	0.9595	0.5388	0.5329	0.5576	0.5337
0.0100	0.9524	0.9502	0.9574	0.9536	0.5397	0.5012	0.5028	0.5383
0.0125	0.9499	0.9508	0.9518	0.9557	0.5040	0.5134	0.5079	0.5015
0.0150	0.9505	0.9522	0.9520	0.9507	0.5216	0.5116	0.5384	0.5107
0.0175	0.9489	0.9497	0.9489	0.9479	0.5149	0.5069	0.5165	0.5317
0.0200	0.9522	0.9485	0.9494	0.9509	0.5133	0.5112	0.5139	0.5113
0.0225	0.9514	0.9519	0.9457	0.9548	0.5072	0.5076	0.5048	0.5151
0.0250	0.9520	0.9512	0.9465	0.9516	0.5086	0.5115	0.5051	0.5179
	------Coverage 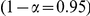 --------				---------Assurance 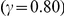 -----------			
0.0050	0.9543	0.9528	0.9532	0.9566	0.8286	0.8531	0.8413	0.8109
0.0075	0.9554	0.9523	0.9551	0.9516	0.8206	0.8051	0.8029	0.8293
0.0100	0.9516	0.9524	0.9560	0.9545	0.8296	0.8019	0.8206	0.8415
0.0125	0.9476	0.9473	0.9508	0.9529	0.8092	0.8226	0.8016	0.8167
0.0150	0.9477	0.9517	0.9511	0.9526	0.8077	0.8028	0.8161	0.8128
0.0175	0.9504	0.9503	0.9502	0.9466	0.8108	0.8170	0.8063	0.8180
0.0200	0.9508	0.9514	0.9504	0.9504	0.8089	0.8050	0.8180	0.8146
0.0225	0.9498	0.9500	0.9460	0.9527	0.7995	0.8131	0.8092	0.8034
	-------Coverage 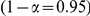 --------				---------Assurance 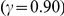 -----------			
0.0050	0.9492	0.9525	0.9527	0.9537	0.9223	0.9104	0.9223	0.9294
0.0075	0.9504	0.9529	0.9526	0.9548	0.9050	0.9165	0.9242	0.9103
0.0100	0.9505	0.9520	0.9518	0.9493	0.9130	0.9054	0.9106	0.9056
0.0125	0.9524	0.9533	0.9512	0.9513	0.9113	0.9093	0.9039	0.9158
0.0150	0.9484	0.9498	0.9492	0.9551	0.8985	0.8999	0.9016	0.9088
0.0175	0.9486	0.9486	0.9510	0.9478	0.9070	0.9023	0.9090	0.9061
0.0200	0.9518	0.9482	0.9495	0.9567	0.9019	0.9011	0.9074	0.9067
0.0225	0.9494	0.9534	0.9509	0.9472	0.8969	0.9041	0.9064	0.9089
0.0250	0.9492	0.9511	0.9533	0.9530	0.9056	0.8986	0.9036	0.9019

d These coverages and these levels of assurance are for sample sizes obtained with the computational Wald procedure (method 2) presented in Table 2, for a CI of 95%, 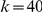 four desired widths (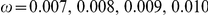), and three values of assurance 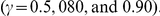

**Table 5:** Simulation study of coverage and assurance for method 1^e^.

	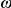	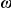
*p*	0.007	0.008	0.009	0.010	0.007	0.008	0.009	0.010
	-------Coverage 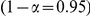 --------				----------Assurance 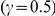 -----------			
0.0050	0.9537	0.9564	0.9532	0.9566	0.5383	0.5426	0.5696	0.5513
0.0075	0.9555	0.9535	0.9543	0.9593	0.5404	0.5303	0.5564	0.5375
0.0100	0.9499	0.9547	0.9527	0.9537	0.5673	0.5402	0.5721	0.5426
0.0125	0.9540	0.9517	0.9513	0.9527	0.5607	0.5776	0.5795	0.5863
0.0150	0.9556	0.9493	0.9550	0.9500	0.5650	0.5945	0.5486	0.5651
0.0175	0.9529	0.9525	0.9554	0.9509	0.5660	0.5968	0.5521	0.5635
0.0200	0.9517	0.9506	0.9552	0.9505	0.5953	0.5836	0.5930	0.5740
0.0225	0.9527	0.9488	0.9516	0.9545	0.5940	0.6096	0.5919	0.5859
0.0250	0.9507	0.9491	0.9487	0.9523	0.6014	0.6093	0.6103	0.5903
	------Coverage 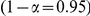 --------				---------Assurance 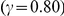 -----------			
0.0050	0.9549	0.9518	0.9526	0.9551	0.8299	0.8509	0.8453	0.8478
0.0075	0.9538	0.9549	0.9529	0.9538	0.8182	0.8563	0.8384	0.8296
0.0100	0.9511	0.9502	0.9505	0.9551	0.8403	0.8336	0.8388	0.8369
0.0125	0.9511	0.9526	0.9547	0.9541	0.8422	0.8602	0.8517	0.8324
0.0150	0.9523	0.9517	0.9537	0.9521	0.8493	0.8456	0.8631	0.8429
0.0175	0.9489	0.9537	0.9471	0.9517	0.8444	0.8534	0.8478	0.8544
0.0200	0.9513	0.9537	0.9537	0.9510	0.8567	0.8593	0.8587	0.8530
0.0225	0.9494	0.9512	0.9512	0.9531	0.8525	0.8427	0.8692	0.8606
	-------Coverage 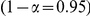 --------				---------Assurance 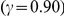 -----------			
0.0050	0.9529	0.9543	0.9522	0.9509	0.9234	0.9112	0.9235	0.9280
0.0075	0.9521	0.9536	0.9507	0.9534	0.9235	0.9140	0.9237	0.9086
0.0100	0.9500	0.9516	0.9527	0.9522	0.9217	0.9107	0.9188	0.9350
0.0125	0.9492	0.9501	0.9547	0.9529	0.9165	0.9263	0.9269	0.9185
0.0150	0.9493	0.9533	0.9535	0.9494	0.9232	0.9284	0.9323	0.9385
0.0175	0.9492	0.9531	0.9505	0.9518	0.9249	0.9355	0.9321	0.9307
0.0200	0.9477	0.9512	0.9486	0.9520	0.9238	0.9402	0.9299	0.9336
0.0225	0.9530	0.9471	0.9478	0.9539	0.9346	0.9380	0.9340	0.9347
0.0250	0.9511	0.9492	0.9504	0.9516	0.9381	0.9371	0.9416	0.9316

e These coverages and levels of assurance are for sample sizes obtained with the exact Clopper-Pearson (method 1) presented in Table 2, for a CI of 95%, 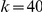, four desired widths (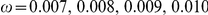), and three values of assurance 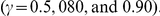

### Comparing the proposed analytic formula with two exact computational procedures using group size *k* = 40

Although the Clopper-Pearson CI is conservative, it is regarded as the gold standard reference method. First the sample size of methods 2 (computational Wald procedure) and 3 (analytic formula Eq. 9) are compared with the sample size resulting from using the exact Clopper-Pearson CI (method 1). For example, when 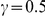 and 0.8, the analytic method (method 3; Eq. 9) underestimates the sample size from 1 to 10 pools (Table 2), while the computational Wald procedure (method 2) underestimates the sample size from 1 to 9 pools with regard to the Clopper-Pearson (method 1) sample size. When 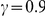, the underestimation is from 3 to 13 pools using the analytic method (method 3; Eq. 9) and from 1 to 10 pools using the computational Wald procedure (method 2). It is important to point out that the level of underestimation increases for bigger values of the proportion (*p*); when the proportion is less than 0.01, the underestimation can be considered negligible because it is less than 5 pools and decreases for smaller values of *p*.

On the other hand, comparing the analytic method (method 3; Eq. 9) with the computational Wald procedure (method 2), the analytic method (method 3; Eq. 9) produces at most 5 pools less than the exact Wald procedure (Table 2), which shows that the difference between these two methods is not important. For the analytic method (method 3; Eq. 9), the level of underestimation can be considered irrelevant when 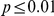 and of little relevance when 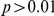, given that the Clopper-Pearson method (method 1) produces a considerable overestimation due to the use of a conservative CI procedure.

Suppose a researcher is interested in estimating *p* for AP maize in the region of Oaxaca, Mexico, where AP maize was reported to be found. With this information and after doing a literature review, it is considered that *p* = 0.01, with a CI of 95%, and *k = 40*, and it is assumed that the final CIW is 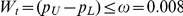. The application of the proposed methods leads to the required number of preliminary pools of 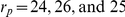, each of size *k* = 40, using the analytic (method 3; Eq. 9), Clopper-Pearson (method 1; Eq.7), and computational Wald methods (method 2; Eq.7), respectively. These sample sizes are contained in the first sub-table of Table 2 (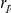 with 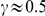, where *k* = 40, *p* = 0.01, and 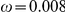).

Realizing that 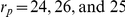 will lead to a sufficiently narrow CI only about 50% of the time, the researcher incorporates an assurance of 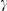 = 0.90, which implies that the width of the 95% CI will be larger than the required width (i.e., 0.008) no more than 10% of the time. From the third sub-table of Table 2 (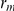 with 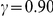), it can be seen that the modified sample size procedure yields the necessary number of pools 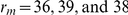 for the analytic method (method 3), Clopper-Pearson method (method 1), and computational Wald procedure (method 2), respectively. Using these sample sizes (36, 39, and 38) will provide 90% assurance that the CI obtained for 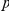 will be no wider than 0.008 units. This sample size is contained in the third sub-table of Table 2 (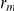 with 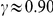, where *k* = 40, *p* = 0.01, and 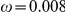).

### An example using the proposed formula (method 3)

In this subsection, we will illustrate the use of the developed formula (Eq. 9) called method 3. Assume that a researcher is interested in estimating *p* and she/he hypothesizes that *p* = 0.02, and wants a CI of 95%, pool size *k = 40*, and a desired error equal to 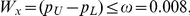 with an assurance level of 99% (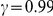). First, it is necessary to calculate 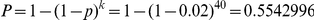, 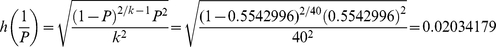, and 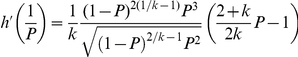 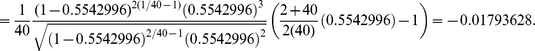 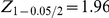 because the CI is 95%, 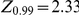 because it is assumed that the assurance level is 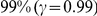, 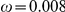, 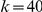. Therefore,
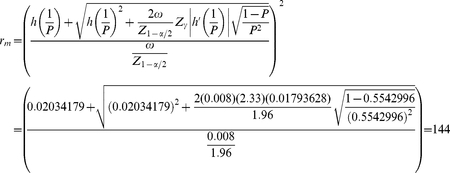
With Eq. (9), the optimum number of positive pools is calculated with a 99% probability that the CI width will be smaller than 0.008, the desired error. Note that for calculating 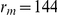, the double precision format was used; otherwise, a slight overestimation would have occurred. It should be pointed out that if 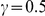, the value of 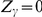 and the required number of pools reduces to Eq. (5), that is, 99 pools.

Appendix S2 provides information for implementing the proposed methods and for obtaining sufficiently narrow CIs for any combination of 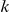, 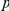, 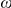, 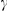, and 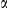 using the R package [28]. The R package computes the sample size using the proposed formula, Eq. (9), and the two proposed computational sample size methods.

### Coverage and assurance levels–simulation study

In this subsection we will examine whether the three sample size procedures [analytic (method 3), computational Wald (method 2) and exact Clopper-Pearson (method 1)] achieve: (1) the coverage probabilities of the nominal (1-α)100% CI used to calculate the CIs, and (2) the nominal levels of assurance, because this sample size formula (Eq. 9) and the two computational methods were derived under the AIPE approach.

For each sample size (number of positive pools, (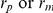) from each combination of 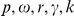 reported in Table 2 and obtained from Equations (7) or (9), we took 40,000 random samples of size 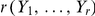, where 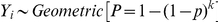, to examine the coverage and assurance levels for each sample size (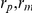). First we obtained the corresponding CI from the 40,000 random samples, and then we counted the proportion of CI that contains the true value of *p*, and the proportion of CI that has a CI width narrower than the desired CI width (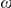). In Table 3, we can see that the coverage of the confidence intervals corresponding to the sample sizes for the analytic method (method 3) obtained from Table 2 is very similar to the nominal level (95%) and in most cases is slightly greater than 95%. These results are not in agreement with other studies that showed that the coverage of small sample sizes using the Wald CI is poor. The Wald CI performed very well here perhaps due to the relatively large sample sizes and also because the parameter 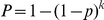 in the cases studied here is around 0.5, which causes less skewing in the distribution of 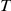; consequently, the normal approximation is better. Also, the coverage of the sample sizes in Table 4 [for the computational Wald (method 2)] and in Table 5 [exact Clopper-Pearson (method 1)] is in most cases slightly greater than the nominal level (95%).

Concerning the level of assurance, we can see in Table 3 [for the analytic procedure (method 3)] that for the three levels studied (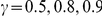) the obtained assurances are smaller than the specified nominal values. The results for 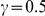 are consistent with the results in Table 1, which indicates that sample sizes with no assurance (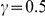) guarantee a desired CI width around 50% of the time and, in most cases, less than 50%. Also, when the assurance is 80% or 90%, the achieved levels of assurance are smaller than the nominal levels. For the computational Wald procedure (Table 4), we can see that the assurance levels in most cases are slightly greater than the specified nominal level (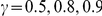). Finally, for the exact Clopper-Pearson procedure (Table 5), the levels of assurance reached are larger than the nominal values in all cases, and we can say that there is an evident overestimation of the specified nominal values (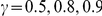).

## Discussion

This paper presented three methods for determining the optimal sample size for estimating the proportion of transgenic plants in a population, assuming perfect sensitivity and specificity, which must be taken into account when designing a study. The proposed methods guarantee that the desired CI width (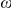) will be achieved with a probability 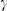, because they take into account the stochastic nature of the confidence interval width. Of the three methods presented, two are computational and one is analytic. According to the Monte Carlo study, the computational Wald procedure (method 2) is the best option because its corresponding coverage and assurance levels are very close to the nominal specified values. On the other hand, the exact Clopper-Pearson procedure (method 1) is conservative (overestimates the required sample size) because the coverage (in most cases) and assurance levels (in all cases) are larger than the nominal values; the analytic procedure (method 3) slightly underestimates the required sample sizes because in most cases the observed levels of assurance are smaller than the nominal values, even though in most cases the coverage reached is slightly greater than the nominal level (95%).

The main advantage of the analytic procedure (method 3) is that a simple formula (Eq. 9) was derived which, within a certain range of *k*, *p*, and 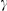, is very precise and produces similar results to the two computational methods proposed. However, the proposed formula underestimated the optimum number of positive pools, mainly for 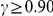, for *k*>75 at *p*>0.01. However, if the number of pools given by the formula (Eq. 9) of the analytic method increases to 6, the resulting sample size will be very close to the computational Wald CI, which produces, on average, 5 pools more than the analytic procedure (method 3).

The three proposed methods are good approximations for determining the optimal sample size under negative binomial group testing, because they were derived using two types of confidence intervals (Wald and Clopper-Pearson). Although the Clopper-Pearson CI is considered the gold standard, its corresponding sample size (method 1) is conservative (overestimates the sample size) and it is not possible to compute it analytically. For this reason, we recommend using the sample size resulting from the computational Wald procedure (method 2). A disadvantage of method 2 is that it does not have an analytic solution.

These methods using group testing are an excellent option under the assumption that AP concentration is low, 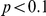. Pool size can be an important consideration, since from an economic perspective, it is always better to have a large pool size and a smaller number of pools than vice versa. However, pool size should be chosen carefully to avoid a high rate of false negatives. On the other hand, an important point to take into account when using the negative binomial group testing sampling method is that the sample size (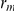) given by Equations (7) and (9) represents the number of positive pools required to stop the sampling and testing process. The sampling and testing process is performed pool by pool using simple random sampling until we find the required number of positive pools (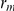). That is, sampling and testing will stop when the number of positive pools, 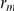, is reached and we need to record the observed data 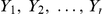, to get the overall number of pools tested 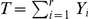.

Note that the sample size formula developed by Montesinos-López et al. [21] under binomial group testing looks similar to those developed in this study; however, here we derived the three procedures under inverse negative binomial group testing sampling, that is, using negative binomial distribution. In the method of Montesinos-López et al. [21], the required sample size is a fixed quantity (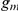: number of pools to study, which represents the number of laboratory tests to be performed); under negative binomial group testing, the number of positive pools (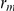) is the quantity that is fixed in advance, whereas the overall number of pools tested is a random variable, because the sampling and testing process stops when the 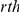 positive pool is found. The methods proposed here give the value of the required number of positive pools (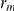).

The R program (see Appendix S2) developed using the R package [28] allows the user to quickly and simply plan the sample size according to her/his requirements or needs using the three proposed methods [the analytic (method 3), exact Clopper-Pearson (method 1) and computational Wald methods (method 2)]. However, if the researcher does not have access to the R program, the best practical solution is the analytic procedure using Eq. (9).

## Supporting Information

Appendix S1 (DOC)

Appendix S2 (DOC)
