# Supplementary material for: Sample Size under Inverse Negative Binomial Group Testing for Accuracy in Parameter Estimation
Source: PLoS One. 2012 Mar 22;7(3):e32250. doi: 10.1371/journal.pone.0032250 (PMC3310835; doi:10.1371/journal.pone.0032250)
Supplement: Appendix S1 — (DOC) [file pone.0032250.s001.doc]

APPENDIX S1

**Result 1** Suppose that $Y_{1}, \ldots, Y_{r}$ is a random sample of size $r$ from a distribution $Geometric\left[P=1-(1-p)^{k}\right]$. Let $h(x)=\sqrt{\frac{\left(1-1/x\right)^{2/k-1}\left(1/x\right)^{2}}{k^{2}}}$ and let $\bar{T}_{r}=\frac{\sum_{i=1}^{r} Y_{i}}{r}$. Then for $P\neq\frac{2k}{2+k}$

that is,

where $\sigma_{r}^{2}=\frac{1-P}{rP^{2}}$, $h\left(\frac{1}{P}\right)=\sqrt{\frac{\left(1-P\right)^{2/k-1}P^{2}}{k^{2}}}$and $h'\left(\frac{1}{P}\right)=\frac{1}{k}\frac{\left(1-P\right)^{2(1/k-1)}P^{3}}{\sqrt{\left(1-P\right)^{2/k-1}P^{2}}}\left(\frac{2+k}{2k}P-1\right).$

Proof

Note that . Then, since $\sigma_{r}^{2}\to0$ if $r\to\infty$, $h(x)=\sqrt{\frac{\left(1-1/x\right)^{2/k-1}\left(1/x\right)^{2}}{k^{2}}}$ is differentiable with respect to $x\in(0,1)$ and $h'\left(\frac{1}{P}\right)=\frac{1}{k}\frac{\left(1-P\right)^{2(1/k-1)}P^{3}}{\sqrt{\left(1-P\right)^{2/k-1}P^{2}}}\left(\frac{2+k}{2k}P-1\right)\neq0$

for $P\neq\frac{2k}{2+k}$, then using the delta method we get, .

**Result 2** Suppose that is a random sample of size from a distribution . Let and let . Then, the smallest integer value such that

is approximately

$$r_{m}=\left(\frac{\sqrt{\frac{\left(1-P\right)^{2/k-1}P^{2}}{k^{2}}}+\sqrt{\frac{\left(1-P\right)^{2/k-1}P^{2}}{k^{2}}+\frac{2\omega}{Z_{1-\alpha/2}}Z_{\gamma}\left|\frac{1}{k}\frac{(1-P)^{2(1/k-1)}P^{3}}{\sqrt{\left(1-P\right)^{2/k-1}P^{2}}}\left(\frac{2+k}{2k}P-1\right)\right|\sqrt{\frac{1-P}{P^{2}}}}}{\frac{\omega}{Z_{1-\alpha/2}}}\right)^{2}$$

Proof

From Result 1 we have that where for $P\neq\frac{2k}{2+k}$ . Let $Z_{\gamma}$ the $\gamma$ quantile of the standard normal distribution. Therefore,

$$\Leftrightarrow P\left[Z\leq\frac{\frac{\omega\sqrt{r_{m}}}{2Z_{1-\alpha/2}}-h\left(\frac{1}{P}\right)}{\sqrt{\left(h'\left(\frac{1}{P}\right)\right)^{2}\frac{(1-P)}{r_{m}P^{2}}}}\right]\approx\gamma\Leftrightarrow\frac{\frac{\omega\sqrt{r_{m}}}{2Z_{1-\alpha/2}}-h\left(\frac{1}{P}\right)}{\sqrt{\left(h'\left(\frac{1}{P}\right)\right)^{2}\frac{(1-P)}{r_{m}P^{2}}}}\approx Z_{\gamma}$$

$$\Leftrightarrow\frac{\omega}{2Z_{1-\alpha/2}}r_{m}-h\left(\frac{1}{P}\right)\sqrt{r_{m}}-Z_{\gamma}\sqrt{\left(h'\left(\frac{1}{P}\right)\right)^{2}\frac{(1-P)}{P^{2}}}\approx0$$

(1.S1)

Note that Eq. (1.S1) has a quadratic form $ax^{2}+bx+c=0$, with , with two solutions given by $x=\frac{-b\pm\sqrt{b^{2}-4ac}}{2a}$. Taking for fixed $\omega$, the desired is given by , in which after replace and we obtain the result and Eq. 9.
