# Supplementary material for: Sample Size under Inverse Negative Binomial Group Testing for Accuracy in Parameter Estimation
Source: PLoS One. 2012 Mar 22;7(3):e32250. doi: 10.1371/journal.pone.0032250 (PMC3310835; doi:10.1371/journal.pone.0032250)
Supplement: Appendix S2 — (DOC) [file pone.0032250.s002.doc]

APPENDIX S2

**Using NBGTSS to implement the analytic formula and the computational methods**

To calculate the appropriate sample size, we have developed the R package NBGTSS, available on the CIMMYT web site (<http://apps.cimmyt.org/english/wps/biometrics/index.htm>) (go to Biometrics and Statistics Unit, then Manuals and Programs). The three methods for computing sample sizes (one analytic and two computational) that are presented and discussed in this article can be implemented using this R package (R Development Core Team, 2007). This appendix provides a brief overview of the NBGTSS functions that can be used to calculate the required sample size. Because NBGTSS is an optional package, it must be loaded during each new R session. Packages in R are loaded with the library() command, which is illustrated with NBGTSS as follows:

R> library (NBGTSS)

To calculate the required sample size with the NBGTSS package, the function rpool() should be used. For example,

rpool(method=“analytic”, *p*=0.02*, k=40, conf.level=*0.95, *width*=0.008, *assurance=*0.99).

In “method” the user needs to specify which method is required to calculate the sample size, that is, method=“analytic” computes the sample size using the proposed formula (method 3, Eq. 9); method=“Clopper-Pearson” calculates the sample size using the Clopper-Pearson CI (method 1), and method=“computational-Wald” calculates the sample size interactively using the Wald CI (method 2). The value $p$ is the population prevalence, *k* is the required pool size, *conf.level* is the confidence level (i.e., 1 -$\alpha$), *width* is the desired CI width, and *assurance* is the desired degree of certainty ($\gamma$) that can be used in the function by specifying the certainty. Implementation of the function above yields the necessary sample size (number of positive pools), which provides 99% assurance that the obtained CI width for *p* will be no wider than 0.008 units. The value of assurance should be at least 0.5 (50%).
